# Well Done

**Published:** 1880-09

**Authors:** 


### WELL DONE.

To do any thing well, there should be a sound mind in a healthy body. There have been men who were perhaps never well, never for an hour enjoyed good health, and yet they lived to purpose, for their deeds are this day exerting a happytying influence on mankind. William the Conqueror was a wheezing asthmatic all his days. Bishop Hall was a martyr to pain as ceaseless as it was severe. Baxter had an infirmity of constitution, and from early youth to the grave, labored under bodily disease, and wearing pains. Calvin scarcely knew in twenty years, what it was to have a well day. No doubt the sufferings of these men aided in molding their characters to a form which the age required. The most we can say of these cases is, that their diseased condition was overruled, and good was brought out of it. What greater good might have resulted had they been men of stalwart constitutions, we may never know, but certain it is, that when we are well, thought is a pleasure, and labor is a pleasure, but when sick, both are a burden, and every thought, and every act, is the result of an effort. We shall never do any thing perfectly, until we get to Heaven, but there, pain, and sickness, and disease can never enter. And if health is needed to enable us to do our duty well in a perfect state, much more is it needed to help us perform our parts well on earth. But whether sick or well, let us do what we may towards fulfilling our duty, and that is all that will be required of us. We can redily see how personal afflictions may humble, and subdue, and sanctify, and thus redound to the good of the individual; but for all that, the great cause of humanity must suffer by it. The Almighty may permit disease, as he permits sin, and we cannot believe that he has any agency in sending either, we bring both on ourselves, but for all that, both may be over-ruled to our good, and his glory.

